# Cytotoxicity and cytokine expression induced by silorane and methacrylate-based composite resins

**DOI:** 10.1590/1678-775720150449

**Published:** 2016

**Authors:** Daniele Lucca LONGO, Francisco Wanderley Garcia PAULA-SILVA, Lucia Helena FACCIOLI, Patrícia Maria GATÓN-HERNÁNDEZ, Alexandra Mussolino de QUEIROZ, Léa Assed Bezerra da SILVA

**Affiliations:** 1 - Universidade de São Paulo, Faculdade de Odontologia de Ribeirão Preto, Departamento de Clínica Infantil, Ribeirão Preto, SP, Brasil.; 2 - Universidade de São Paulo, Faculdade de Ciências Farmacêuticas de Ribeirão Preto, Departamento de Análises Clínicas, Ribeirão Preto, SP, Brasil.; 3 - Universitat de Barcelona, Facultat d’Odontologia, Department d’Odontopediatria, Barcelona, España.

**Keywords:** Dental materials, Composite resins, Cytotoxicity, Cytokines

## Abstract

The successful use of composite resins in Dentistry depends on physicochemical properties, but also on the biological compatibility of resins, because of the close association between pulp and dentin.

**Objective:**

The aim of this study was to evaluate cytotoxicity and cytokine production induced by light-cured or non-light-cured methacrylate-based and silorane composite resins in RAW 264.7 macrophages.

**Material and Methods:**

Cells were stimulated with the extracts from light-cured or non-light-cured composite resins. After incubation for 24 h, cytotoxicity was assessed with the lactate dehydrogenase (LDH) and methyl thiazolyl tetrazolium (MTT) assays, and total protein was quantified using the Lowry method. TNF-α detection was examined with an enzyme-linked immunosorbent assay (ELISA) conducted with cell supernatants after cell stimulation for 6, 12, and 24 h. Data were analyzed using one-way analysis of variance (ANOVA) and Tukey’s *post hoc* test (α=0.05).

**Results:**

Kalore^TM^ and Filtek^TM^ Silorane were cytotoxic with or without light curing (p<0.05) after 24 h of incubation. Kalore^TM^ stimulated the early production of TNF-α in comparison with control (p<0.05), whereas Filtek^TM^ Silorane did not affect TNF-α levels after 6 and 12 h (p>0.05). However, after 24 h Filtek^TM^ Silorane inhibited the production of TNF-α (p<0.05).

**Conclusions:**

Kalore^TM^ and Filtek^TM^ Silorane were cytotoxic regardless of light curing. The extract obtained from Kalore^TM^ after 15 days of incubation stimulated the production of TNF-α, unlike that obtained from Filtek^TM^ Silorane.

## INTRODUCTION

The main components of composite resins include the organic matrix; filler particles; the bonding agent, which connects the filler to the organic matrix; the activator system, which initiates polymerization; pigments, which impart the compound with colors similar to those of teeth; and polymerization inhibitors, which increase the useful life and working time of the material^1,16^. Composite resins are dental materials commonly used to restore the structural integrity and function of teeth affected by caries, erosion, fracture or attrition^11^.

The effective application of resin materials heavily depends on their physicochemical properties. However, clinical success ultimately depends on the biological compatibility of the resins, because of the close relation between pulp and dentin^27^. The filler material does not seem to affect biological compatibility, which mostly depends on the organic components of composite resins^5^. These components include monomers, such as bisphenol A-glycidyl methacrylate (Bis-GMA), triethylene glycol dimethacrylate (TEGDMA), urethane dimethacrylate (UDMA), 2-hydroxyethyl methacrylate (HEMA), and bisphenol A-ethoxylated dimethacrylate (Bis-EMA)^4,16^. Monomers that are not polymerized are released from conventional methacrylate-based composite resins and have been associated with genotoxicity^5,8,9,22,25^, estrogenicity^5,22,26^, changes in the immune system^10,17^, hypersensitivity, cytotoxicity^3,5,8,29^, and the production of reactive oxygen species^3,21^.

The use of low shrinkage monomers and high molecular weight has improved significantly the composite resins, although mechanical and chemical issues still remain, specifically regarding the polymerization shrinkage effects^15,18^. New methacrylate-based composite resins have been developed with changes in the composition, structure, and nature of polymerization, such as the resin Kalore™ (GC FUJI, Kasugai, Japan), which uses the high molecular weight Dupont monomer DX-511^4^. Conversely, the composite Filtek™ Silorane, brought to market by 3M ESPE (Seefeld, Germany), does not have a methacrylate monomer as its organic matrix, instead, it uses siloxane and oxirane molecules^28^. To date, no studies have evaluated the cytotoxicity and the induction of pro-inflammatory molecules mediated by these composite resins in RAW 264.7 macrophages.

Therefore, this study assesses the cytotoxic effects and the production of cytokines induced by light-cured or non-light-cured methacrylate-based and silorane composite resins in RAW 264.7 macrophages.

## MATERIAL AND METHODS

### Extract preparation

This study was carried out according to the standards of the International Organization for Standardization (ISO) no. 10993-5:2009^7^.

In the absence of artificial light, composite resins were removed from their tubes with a sterile spatula #1, placed on sterile paper, and weighed (PG 503-S, Mettler Toledo^®^; Toledo, Ohio, USA). Cell viability and cytokine production were evaluated after the exposure of the cells to the following experimental groups: Kalore^TM^and Filtek^TM^Silorane (light-cured and non-light-cured) (Table 1).

**Table 1:** Compositions of KaloreTM and Filtek™ Silorane resins, according to the information provided by the manufacturers

Composite Resin	Manufacturer/Color	Particle	Organic Matrix	Inorganic Load (C.I.)	% C.I.
Kalore™	GC FUJI/A3	Nanohybrid	DX-511 UDMA dimethacrylatenonspecific comonomers	Glass Fluoroaminosilicate (silica and silicon dioxide)	82
Filtek™ Silorane	3M ESPE/A3	Microhybrid	Siloxane Oxirane	Quartz Yttrium fluoride	76

Light curing was performed using a light-emitting diode (LED) device (RADII-CAL SDI Limited; Bayswater, Victoria, Australia) with a light intensity of 1,200 mW/cm^2^ and a wavelength of 430-480 nm. The polymerization process used for the composite resins followed the manufacturer’s instructions, 20 seconds for each increment of composite resin (20 mg). For the groups of resins that were not light cured, the materials were kept in the dark until the moment of use.

To extract their components, composite resins were placed in 12-well plates and properly identified, and 3 mL of culture medium (DMEM) containing 500 µl of gentamicin (10 mg/mL; Gibco; Grand Island, NY, USA) and 5 mL of penicillin (100 µg/mL; Gibco; Grand Island, NY, USA) and streptomycin (100 µg/mL; Gibco; Grand Island, NY, USA) were added, so that the material was fully covered by this solution for 30 min. The solution was then removed with a serological pipette and each well of the plate was washed with 3 mL of PBS and filled with 3 mL of DMEM. Composite resins were weighed and the extracts 20-80 mg of resin per mL of DMEM were obtained after 15 d of incubation.

### Cell culture

The RAW 264.7 murine macrophage cell line was obtained from the American Type Culture Collection (ATCC; Rockville, MD, USA). The cells were grown in DMEM medium supplemented with 10% fetal bovine serum and 1% gentamicin (DMEM-c). After the formation of a monolayer, cells were harvested with plastic cell scrapers and centrifuged at 1,500 rpm for 10 min at 10°C (Eppendorf; Hamburg, Hamburg, Germany). After centrifugation, supernatants were discarded and 10 mL of DMEM-c was added to each tube of cells. The total number of cells was counted and the viability was determined in a Neubauer chamber (BOECO Germany; Hamburg, Hamburg, Germany) using Trypan blue (Gibco; Grand Island, NY, USA). Cells were then plated in 96-well culture plates (Cell Wells – 25,820, Corning Glass Works; New York city, NY, USA) at a density of 1x10^5^ cells/well and incubated overnight in DMEM-c in an incubator with a moist atmosphere of 5% CO_2_ and 95% air at 37°C. Cells were then incubated with the extracts of composite resin for 24 h.

### Lactate dehydrogenase (LDH) assay

The cytotoxicity of the resin extract in RAW 264.7 macrophages was evaluated through the level of LDH released in the cell supernatant following cell lysis using the CytoTox96^®^ non-radioactive cytotoxicity assay (Promega Corporation; Madison, WI, USA). The absorbance was measured at 490 nm with a spectrophotometer (mQuanti, Bio-Tek Instruments, Inc.; Winooski, VT, USA). LDH levels were expressed as percentages of the LDH levels observed in control cultures.

### MTT assay

Cell viability was evaluated using the 3-(4,5-dimethylthiazol-2-yl)-2,5-diphenyltetrazolium bromide (MTT) colorimetric assay (Sigma-Aldrich)^25^. The cells were incubated with the extracts of composite resin for 24 h, then the cultures were incubated with 5% MTT in RPMI for 3 h. Subsequently, 50 mL of 20% sodium dodecyl sulfate (SDS) in 0.01 M HCl were added to each well and maintained at room temperature until the precipitate completely solubilized. Absorbance was measured at 570 nm with a spectrophotometer (mQuanti) and was directly proportional to cell viability. The cytotoxicity of the composite resins was expressed as percentages of the cytotoxicity observed in non-stimulated control cells.

### Total protein quantification

Total protein quantification was performed using the Lowry method (Bio-rad DC Protein assay). Absorbance was measured at 750 nm with a spectrophotometer (mQuanti). Data are expressed as mg/mL obtained based on a standard curve using bovine serum albumin (BSA).

### TNF-α detection

The concentration of TNF-α in culture supernatants was quantiﬁed by ELISA using specific purified and biotinylated antibodies and cytokine standards, according to the manufacturers’ instructions (R & D Systems; Minneapolis, MN, USA). The optical densities were measured at 450 nm by a microplate reader. The cytokine concentrations were determined using a standard curve established with the appropriate recombinant cytokine, and are expressed in pg/mL.

### Statistical analyses

Data represent the mean±SEM. Statistical variations were determined by one-way ANOVA and Tukey’s test. Values of *p*<0.05 were considered to be signiﬁcant.

## RESULTS

### Methacrylate-based and silorane composite resins are cytotoxic

**Figure 1:**
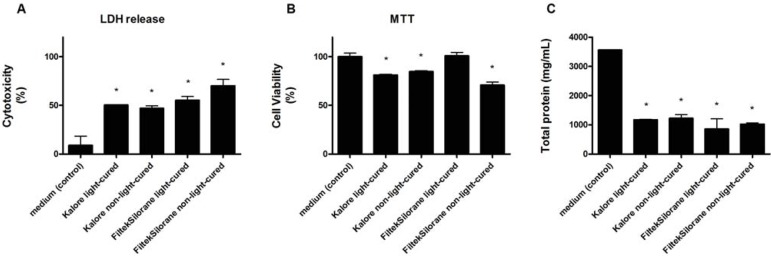
Effects of different composite resin extracts (KaloreTM and Filtek™ Silorane, light-cured and non-light-cured) on murine RAW 264.7 macrophage cultures, as shown by the LDH assay (A), MTT assay (B), and total protein quantification (C), after 24-hour incubation. Statistically significant differences (p<0.05) in relation to the control group (macrophages incubated in culture medium only) are indicated by *

Methacrylate-based and silorane composite resins induced cell lysis and LDH release into the cell supernatant regardless of whether resins were light cured or not (Figure 1A). Intracellular dehydrogenase activity, measured by MTT, was reduced by the methacrylate-based composite resin with or without light curing (*p*<0.05). A similar effect was observed when cells were exposed to the extract of non-light-cured silorane composite resin, but not with the light-cured resin (Figure 1B). Total protein quantification indicated that methacrylate-based and silorane composite resins inhibited cell activity (*p*<0.05, Figure 1C).

### Macrophage TNF-α production is stimulated by methacrylate-based composite resin and inhibited by silorane

**Figure 2:**
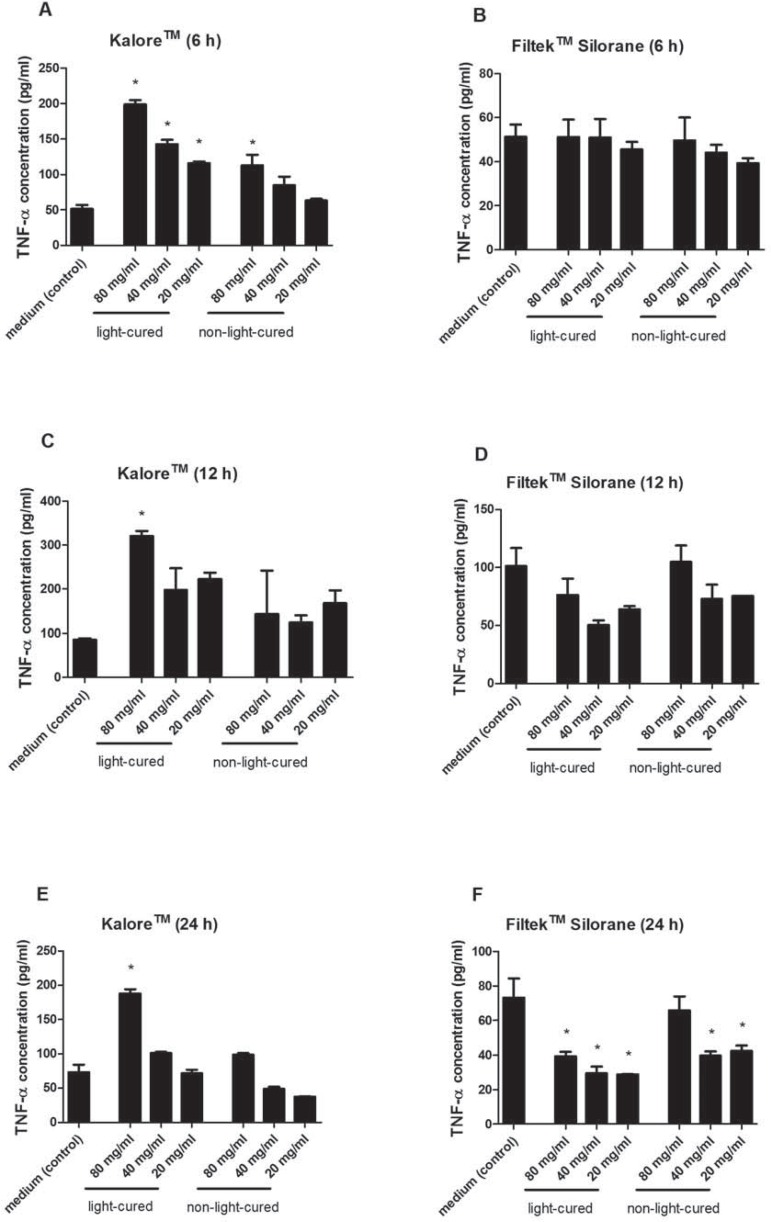
Effects of different composite resin extracts (KaloreTM and Filtek™ Silorane, light-cured and non-light-cured) on the release of TNF-α by cultured murine RAW 264.7 macrophages, as assessed by ELISA after incubation periods of 6 (A, B), 12 (C, D), and 24 (E, F) h. Statistically significant differences (p<0.05) in relation to the control group (macrophages incubated in culture medium only) are indicated by *

The extract of the methacrylate-based composite resin stimulated the early production of TNF-α in comparison with the control treatment (*p*<0.05, Figure 2A and 2C). The silorane composite resin extract did not elicit statistically significant differences in the production of TNF-α relative to the control after 6 and 12 h of incubation (*p*>0.05, Figure 2B and 2D). Interestingly, after 24 h of incubation, the methacrylate-based composite resin sustained TNF-α release whereas the silorane resin extract inhibited the production of TNF-α compared with the control (*p*<0.05, Figure 2E and 2F).

## DISCUSSION

Methacrylate-based and silorane composite resins were cytotoxic with or without light curing as indicated by LDH release and total protein quantification. Extracts of methacrylate-based composite resins stimulated the production of TNF-α after 6 h of incubation. On the other hand, silorane resin extract inhibited the production of TNF-α after 24 h of incubation.

Non-light-curing or incomplete light-curing of methacrylate-based composites results in the release of resin matrix components, called residual monomers, i.e., unpolymerized monomers^2,21^. The release of these non-light-cured resin materials has been associated with various adverse effects^3,5,8-10,17,21,22,25,26,29^. Regarding cytotoxic effects specifically, it is known that isolated monomers cause several biological effects on cells, such as damage to the cell membrane, inhibition of metabolic enzyme activity, cell-cycle delay and interruption, gene mutation, DNA breakage and apoptosis after reduction of GSH (glutathione) via oxidative stress^22^. Several previous studies have investigated the toxicity of monomers in isolation in various cell types^3,5,8,22,29^. However, a recent study using RAW 264.7 macrophages showed that co-exposure to TEGDMA and both types of filler particles, Nanosilica and Quartz, resulted in an additive attenuation of the LPS-induced IL-1β release, but cellular viability and TNF-α release were not significantly affected^13^. On the other hand, the assessment of these isolated monomers does not faithfully reproduce the procedure for the routine use of these materials in the clinic, where they are applied in the polymerized form, i.e., in the form of composite resin.

Our findings revealed that the methacrylate-based composite was cytotoxic *in vitro* in RAW 264.7 mouse macrophages. Similarly, another study showed the biological effects of metacrylate-based composite resins, including possible changes to the DNA of skin cells in class V restorations^23^, and the authors concluded that the resin may cause cellular damage in fibroblasts^24^. However, the silorane composite resin did not affect intracellular mitochondrial dehydrogenase activity, except when it was not light-cured. The difference between the observed cytotoxicity in this study, and the satisfactory compatibility obtained in other studies by our group^19,20^ may result from complete or incomplete curing of the composite resin, respectively. Although we observed a cytotoxic effect of the silorane composite resin, this was not reflected on the MTT assay. Likewise, another study suggested the non-toxic nature of the silorane composite resin in human fibroblasts treated with Filtek P90, which showed only an insignificant decrease in cell proliferation in 24 h and 48 h^12^.

The macrophage lineage, like many cells of the immune system, plays a role in innate immunity-related functions and in the production of inflammatory mediators, such as TNF-α^14^. The metacrylate-based composite resin sustained TNF-α production for up to 24 h of incubation (80 mg/mL), although lower concentrations of the extracts (20-40 mg/mL) stimulated only the early production of TNF-α (6 h). This contrast can be explained by cell death, which decreases the production of TNF-α.

Finally, the silorane composite resin did not induce TNF-α after 6 and 12 h, but inhibited it after 24 h. The decrease in TNF-α levels induced by unpolimeryzed silorane composite resin can also be associated with cytotoxicity. However, that reduction also happened when the resin was polymerized, and in parallel with unchanged cell viability. Thus, the silorane composite resin induced an anti-inflammatory response. Another study revealed that the silorane composite resin had a negative effect on TNF-α levels^6^.

We conclude that Kalore^TM^ and Filtek^TM^ Silorane were cytotoxic regardless of light curing. Interestingly, Kalore^TM^ stimulated the production of TNF-α, unlike Filtek^TM^ Silorane.
